# Barriers and enablers to shared decision-making in assessment and management of risk: A qualitative interview study with people using mental health services

**DOI:** 10.1371/journal.pmen.0000157

**Published:** 2024-11-13

**Authors:** Nafiso Ahmed, Lisa Reynolds, Sally Barlow, Kathleen Mulligan, Nicholas Drey, Alan Simpson

**Affiliations:** 1 Division of Psychiatry, University College London, London, England, United Kingdom; 2 Strategy and Partnerships, Oxford Health NHS Foundation Trust, Oxford, England, United Kingdom; 3 Centre for Mental Health Research, School of Health & Psychological Sciences, City St George’s, University of London, London, England, United Kingdom; 4 Department of Health Services Research and Management, School of Health & Psychological Sciences, City St George’s, University of London, London, England, United Kingdom; 5 East London NHS Foundation Trust, London, England, United Kingdom; 6 Department of Nursing, School of Health & Psychological Sciences, City St George’s, University of London, London, England, United Kingdom; 7 Florence Nightingale Faculty of Nursing, Midwifery and Palliative Care, and Health Service and Population Research, Institute of Psychiatry, Psychology and Neuroscience King’s College London, London, England, United Kingdom; Association for Socially Applicable Research (ASAR), INDIA

## Abstract

The assessment and management of risk are fundamental to mental health care provision and are considered high-priority tasks by professionals worldwide. Clinical guidance recommends for risk to be identified and managed collaboratively with the individual, but studies indicate that this may not be happening in practice. The aim of this study was to identify the barriers and enablers to collaborative risk assessment and management based on shared decision-making from service users’ perspectives. A qualitative approach using semi‐structured interviews was employed. The Theoretical Domains Framework for behaviour change, which consists of fourteen theoretical domains that have been found to influence behaviour, informed data collection and analysis. Thirteen service users living with severe mental illness took part in an interview. The majority of participants reported not having been involved in the identification of their risk and most were unaware of the information included in their risk management plan. Perceived barriers to involvement were power dynamics between professionals and service users, difficulty talking about sensitive risk topics, and the emotional impact of engaging in these discussions. Perceived enabling factors for involvement included the possibility of gaining a better understanding of risk issues, and discussion about risk enhancing the individual’s ability to maintain their own well-being and safety. Most participants expressed a willingness to be involved in shared decision-making and believed that their friends and family would enable them to be involved. The findings of this study offer valuable insights for targeting behaviour change in future intervention design that seeks to increase shared decision-making in risk assessment and management with individuals with severe mental illness.

## Introduction

In most psychiatric settings worldwide, assessing and managing risk are integral to delivering safe services and, thus, considered high-priority tasks by professionals [1, 2]. Risk, in a mental health context, is commonly classified into three broad categories: (1) risk to self, including suicide and self-harm; (2) risk to others, including violence and aggression; or (3) vulnerabilities, including side-effects, exploitation, and discrimination [3, 4].

There are three methods for assessing risk in mental health care: unstructured clinical judgment, actuarial tools, and structured clinical judgment. In unstructured clinical judgment, mental health professionals rely on their intuition and expertise to identify risks [5]. The actuarial approach involves the use of statistical models or structured tools to predict the likelihood of adverse outcomes [6]. Due to limitations in using either of these approaches including risk of errors, poor predictive accuracy of risk tools, and lack of individualisation of clinical judgment [5, 7], clinical guidelines recommend using the combined approach of structured clinical judgement [8]. The risk management plan is developed from the risk assessment, and consists of a list of strategies aimed at preventing or minimising the risks from occurring [3, 6].

The assessment of risk is performed at various stages of mental health care, from initial assessment through to discharge. To identify historical risks and immediate safety concerns, an initial risk assessment is performed at point of admission or as part of referral to a new service [3, 9]. The assessment is reviewed at specific time points associated with increased risk, such as discharge, to enable professionals to evaluate whether previous concerns have been minimised, and plan for transfer between services particularly when the level of security and monitoring provided changes i.e., discharge from inpatient hospital to community mental health services [10]. Risk assessment and management are crucial for safety and decision-making during a crisis or in crisis planning [1], but to effectively monitor any changes in the service users’ situation and to prevent crisis, risk should be reviewed regularly as part of routine clinical practice [3, 11].

Clinical guidelines and healthcare policies mandate incorporating risk assessment and management into routine care [3, 8], emphasising their role in enhancing patient safety, supporting clinical decision-making, and transition between care settings [3, 12], such as inpatient care and community settings [10]. However, evidence of the effectiveness of risk assessment tools in reducing harm remains limited. Research suggests that these tools are often ineffective in predicting suicide and self-harm [13]. Reflecting this, the National Institute for Health and Care Excellence (NICE) guidelines recommend against using risk assessment tools to predict suicide, repetition of self-harm, or determine access to treatment or discharge decision [14]. Instead, they advocate for an assessment that focuses on the persons needs and prioritise their immediate and long term psychological and physical safety.

Research supports the potential benefits of therapeutic person-centred risk assessment in reducing suicide [15], and the positive effects of structured clinical judgement in mitigating violence [12, 13]. This approach aligns with good practice guidelines that call for collaborative assessment and management of risk, with an emphasis on sharing risk documents with all relevant parties, including the service user [3]. Such collaboration may increase the identification and understanding of a wider range of safety concerns [16, 17], enhance engagement with clinicians and services [15], and enable the development of a more person-centred risk management (RM) plan that meets the individual’s needs [11].

Shared Decision-Making (SDM) has been well-researched in physical health care, with numerous frameworks and tools available to aid implementation [18]. However, despite being included in international healthcare policy [19], it has been less developed in mental health care with most psychiatric SDM models and interventions focusing on psychopharmacological decision-making or medication management [20–22]. Stacey, Felton [23] propose a SDM model, which they developed in collaboration with service users, carers and professionals, specifically for mental health contexts. They posit that SDM occurs when all participants are informed, involved, and influential. However, they recognise the challenges in power-sharing in decision-making with mental health service users, given complexities related to mental capacity and the imperative to manage risks, including the risk of suicide and harm to others. Nonetheless, methods, such as advance directives or SDM tools, can be used to determine a service user’s preferred role in decision-making or support their influence before any potential loss of capacity [24].

A recent review indicates that SDM is not routinely implemented with people living with severe mental illness [25], and studies have found that service users are often unaware that a risk assessment has taken place or of the contents of their management plan [4]. A lack of service user involvement in the identification of risk has been reported in studies conducted in both community and forensic services [17, 26]. For instance, a study that interviewed 13 service users considered to pose a risk to others found that participants lacked knowledge of and involvement in risk assessment procedures [27]. The service user’s preference for involvement or perceptions of their role in decision-making around risk has also been highlighted in studies. Coffey et al. [4] found that some service users receiving community mental services viewed risk assessment as a professional priority and considered their role in risk discussions as passive recipients rather than equal contributors on a shared basis. Similarly, a recent study conducted within forensic mental health settings found that most service user participants perceived risk assessment as a tool to serve the professional rather than to aid them in their recovery [28].

The authors of the present study conducted a synthesis of evidence on mental health professionals’ perceived barriers and enablers to SDM in risk assessment and management [29]. Our review revealed several factors that may hinder the implementation of SDM, including professionals maintaining responsibility for risk, challenges related to mental capacity, difficulty in having conversations about serious risks such as suicidality and negative beliefs about consequences. This included fear of causing the service user distress, to disengage from care or feel stigmatised, and worry about potential blame for adverse outcomes. On the other hand, facilitating factors included therapeutic relationships supporting discussion about risk, and professionals’ appreciation for SDM [29]. A systematic review of studies reporting on service users’ perspectives of helpful risk management practices within mental health services further supports the benefit of the therapeutic relationship and trust in facilitating discussion about risk [30].

To date, no previous study has reported on service users’ perceived barriers and enablers to SDM in risk assessment and management. Further research may inform clinical practice and help researchers develop interventions that can be tested. The current study aims to explore service users’ experiences and perceived barriers and enablers to shared decision-making in risk assessment and management. The specific research questions are:

1. How do mental health services users experience risk assessment and management?
2. Utilising behaviour change theory, what do mental health service users perceive to be the barriers and enablers to SDM in risk assessment and management?

## Methods

### Design

We employed a qualitative approach utilising semi-structured interviews informed by theories of behaviour change, described below. A qualitative research design was used to allow for in-depth exploration and analysis of individuals’ experiences of shared decision-making in risk assessment and management. This study was part of a PhD thesis completed at City, University of London’s Centre for Mental Health Research. We follow the Standards for Reporting Qualitative Research (SRQR) 21-item checklist [31]. The SRQR checklist can be found in S1 Appendix.

### The Theoretical Domains Framework

The Theoretical Domains Framework (TDF) is a comprehensive framework designed to identify and understand the factors influencing behaviour change [32]. Developed to enhance the accessibility and utility of behaviour change theory for researchers and practitioners, the TDF provides a consolidated framework that integrates constructs from multiple behaviour change theories. The TDF consists of 14 theoretical domains [33, 34], outlined in Table 1, that have been found to influence behaviour. These domains cover a wide range of potential influences on behaviour, which may act as barriers and/or enablers, from individual beliefs and skills to social influences and environmental factors [32, 35]. Using the TDF can help researchers systematically identify the barriers and enablers for the target behaviour, and these insights can then be used in designing tailored interventions targeting these domains. For example, an educational intervention may be implemented if "Knowledge" is identified as a barrier. [36]. In the present study, the TDF was utilised to identify barriers and enablers for increasing SDM in risk assessment and management with individuals with severe mental illness. Cane et al’s (2012) validated version of the framework was used in the design of this qualitative study [33].

**Table 1:** Definitions for each of the 14 TDF domains based on Cane et al, 2012 [33].

Domains	Definition	Constructs
**1. Knowledge**	An awareness of the existence of something	Knowledge (including knowledge of condition /scientific rationale), Procedural knowledge, Knowledge of task environment
**2. Skills**	An ability or proficiency acquired through practice	Skills, Skills development, Competence, Ability, Interpersonal skills, Practice, Skill assessment, Coping strategies
**3. Professional role and identity**	A coherent set of behaviours and displayed personal qualities of an individual in a social or work setting	Professional identity, Professional role, Social identity, Professional boundaries, Professional confidence, Group identity, Leadership, Organisational commitment
**4. Beliefs about capabilities**	Acceptance of the truth, reality, or validity about an ability, talent, or facility that a person can put to constructive use	Self‐confidence, Perceived competence, Self‐efficacy, Perceived behavioural control, Beliefs, Self‐esteem, Empowerment, Professional confidence
**5. Optimism**	The confidence that things will happen for the best or that desired goals will be attained	Optimism, Pessimism, Unrealistic optimism, Identity
**6. Beliefs about consequences**	Acceptance of the truth, reality, or validity about outcomes of a behaviour in a given situation	Beliefs, Outcome expectancies, Characteristics of outcome expectancies, Anticipated regret, Consequents
**7.Reinforcement**	Increasing the probability of a response by arranging a dependent relationship, or contingency, between the response and a given stimulus	Rewards (proximal / distal, valued / not valued, probable / improbable), Incentives, Punishment, Consequents, Reinforcement, Contingencies, Sanctions
**8. Intentions**	A conscious decision to perform a behaviour or a resolve to act in a certain way	Stability of intentions, Stages of change model, Trans-theoretical model and stages of change
**9. Goals**	Mental representations of outcomes or end states that an individual wants to achieve	Goals (distal / proximal), Goal priority, Goal / target setting, Goals (autonomous /controlled), Action planning (with relation to their intention to implement
**10. Memory, attention and decision processes**	The ability to retain information, focus selectively on aspects of the environment and choose between two or more alternatives	Memory, Attention, Attention control, Decision making, Cognitive overload / tiredness
**10. Memory, attention and decision processes**	Any circumstance of a person’s situation or environment that discourages or encourages the development of skills and abilities, independence, social competence, and adaptive behaviour	Environmental stressors, Resources / material resources, Organisational culture /climate, Salient events / critical incidents, Person x environment interaction, Barriers and facilitators
**12. Social influences**	Those interpersonal processes that can cause individuals to change their thoughts, feelings, or behaviours	Social pressure, Social norms, Group conformity, Social comparisons, Group norms, Social support, Power, Intergroup conflict, Alienation, Group identity, Modelling
**13. Emotions**	A complex reaction pattern, involving experiential, behavioural, and physiological elements, by which the individual attempts to deal with a personally significant matter or event	Fear, Anxiety, Affect, Stress, Depression, Positive / negative affect, Burn‐out
**14. Behavioural regulation**	Anything aimed at managing or changing objectively observed or measured actions	Self‐monitoring, Breaking habit, Action planning (with relation to monitoring their habits)

### Researcher characteristics and reflexivity

The first author (NA) independently interviewed all research participants. NA is a Black female trained in carrying out sensitive interviews both as a researcher and through her work with the National Health Service (NHS) and voluntary sector organisations supporting individuals with severe mental illness. Individuals who took part in the interviews did not know NA, and she was not part of their care team. Prior to the interviews, a systematic review exploring mental healthcare professionals’ views of SDM in risk assessment and management [29] was conducted by the authors of this study. This work provided NA with some insight into potential barriers and enablers to SDM from the perspective of mental health professionals, which she was aware could impact interviewing and theme development. Therefore, to reduce bias, a senior author (AS) read the first few transcripts and provided feedback on her interviewing skills. NA kept a reflective diary, which was discussed in supervision meetings with the wider team (AS, LR, SB). Data analysis involved more than two researchers and included both reliability and validity checks (see data analysis).

### Participants

We recruited service users from two Community Mental Health Teams (CMHT) and an Early Intervention Service (EIS) in one NHS trust in England, serving an inner-city, diverse population with high levels of deprivation.

Participants were eligible to take part in the study if they were aged 18 or over, self-reported as being diagnosed with a severe mental illness, including psychotic disorders, schizophrenia, bipolar, and major depression, and were able to provide informed consent. Participants also needed to have been receiving care or treatment from a Community Mental Health Team (CMHT) or Early Intervention Services (EIS) under the Care Programme Approach (CPA) for at least six months. We decided on a duration of six months under CPA, as we believed that this would allow time for an initial RA and RM plan to be formulated for individuals new to services. Risk assessment and management remain an intrinsic part of the care planning process, and the present Community Mental Health Framework recommends for professionals to adopt a personalised approach to managing risk [37]. Due to the financial constraints of hiring an interpreter, only participants who spoke an adequate level of English were eligible to take part in an interview.

### Data collection

#### Procedure

In each setting, the clinical teams’ administrator provided a list of service users under the care of the selected CMHT/EIS and subject to the CPA. The list included key population characteristics, including the name of their care coordinator, the responsible psychiatrist, the date their care plan was last reviewed, and whether an interpreter was required. We asked the responsible psychiatrist to screen the list and exclude service users experiencing a mental health crisis or currently in hospital. The number of service users excluded varied by team, ranging from 0–20 people. Reasons for exclusion included the person being in hospital, non-engagement, e.g., whereabouts unknown, moved away, or the person had recently died.

From the remaining lists, we employed a stratified random sampling strategy to select a sample of service users to interview. This sampling strategy ensures that all participants have an equal chance of selection [38]. A letter of invitation, a participant information sheet, an expression of interest form and a prepaid addressed return envelope were posted to the selected service users. The participants who returned the completed expression of interest form were contacted via phone by the first author (NA), who provided details of the study, answered any questions, and arranged a date and time for the interview. Participants were given at least 24 hours to consider their decision and encouraged to discuss it with family, friends, advocates, or staff if they wished. We waited six weeks after each mailout before posting further invitations to new participants.

Eight batches of service user invitations were posted (EIS = 4 and CMHT = 4), and recruitment continued for eight months (February to October 2017) until the study ended. In addition to recruiting via the lists provided by the team, the receptionist placed advertisements on the notice boards in the CMHT/EIS centre. Service users who saw the advertisement and were interested in taking part could contact the lead researcher (NA), who would check that they met the eligibility criteria and start the process outlined above. The sample size was calculated using the principles for deciding saturation for theory-based interviews [39]. These principles recommend conducting a minimum of 10 interviews for initial analysis, followed by an additional three interviews until no new themes are identified from the data (stopping criterion).

The interviews, lasting between 8 and 58 minutes, were conducted by NA in a private office space at the CMHT/EIS site. All interviews were conducted in person and on a one-to-one basis. Interviews were recorded using a digital audio device and transcribed verbatim professionally (the transcriber signed a confidentiality agreement). NA checked all transcripts against original recordings for accuracy and removed participants’ names and identifying details. Demographic information was collected from each participant using a brief questionnaire adapted from a previous study of care planning [40]. Service users who participated in an interview received a remuneration of £10 in appreciation of their time.

### Ethical approval

Ethical approval was granted by the NHS Health Research Authority Research Ethics Committee London—Camden & Kings Cross (16/LO/1918). A substantial amendment was submitted to increase the sample size, to include the second CMHT site, and to obtain approval to advertise on the notice boards in the CMHT/EIS centre. The same research ethics committee granted a favourable ethical opinion of the amendment.

Participants were provided with detailed written information outlining the study’s purpose and their rights and asked to provide written consent by signing a form. Participants were made aware of the conditional nature of confidentiality, that is, the information they disclosed being treated in confidence except where there are serious concerns for the safety of the participant or a member of the public. At the end of the interview, participants were all provided with information about accessing emotional support in case any of the questions caused them to feel distressed.

### Study material

The first part of the interview schedule asked participants preliminary questions about their experience with risk and whether anyone had ever discussed risk or safety with them. Service users who described being involved continued to the theory-informed interview questions that were structured using the 14 domains of the TDF (see S2 Appendix for the interview schedule).

Service users who reported not having been involved in assessing or managing their risks (or were unsure) were asked hypothetical interview questions that incorporated the TDF less rigidly and contained fewer questions. The hypothetical questions explored participants’ views about not being involved in risk assessment and management, if they would have liked to be involved, and the factors that may have influenced them being involved. Interview questions were substantiated with follow-up questions to prompt participants to elaborate.

The interview schedule did not directly refer to the concept of SDM, as we wanted to improve accessibility. Instead, a model that divides the concept of SDM into three components, i.e., informed, involved, and influential, was applied [23]. Interview questions were worded using these three components.

### Service user and carer involvement

The Service User and Carers Group Advising on Research (SUGAR), a group of mental health service users and carers established to provide advice to researchers at City, University of London [41], were involved in the study design and development of the interview schedule.

SUGAR members were asked for their thoughts about the study’s design and provided a copy of the draft interview schedule to review. Feedback on the content and clarity of the schedule included whether crisis and contingency planning would be considered, and the process of risk assessment and management explained. Feedback on study design and procedure included whether service users using early intervention services would be interviewed, as this setting was not part of the original research proposal. To address these concerns, we included a question on crisis and contingency planning, a definition of risk assessment and management, and we also decided to include an EIS setting.

In addition, NA carried out a role-play of a service user interview with a senior author (AS) in front of the SUGAR group. NA played the role of the interviewer, and AS answered the interview questions from a service user’s perspective. This pilot exercise provided an opportunity to test and receive feedback on her interviewing skills, and the questions. The feedback from the group and how this was addressed can be found in S3 Appendix.

### Data analysis

**Fig 1:**
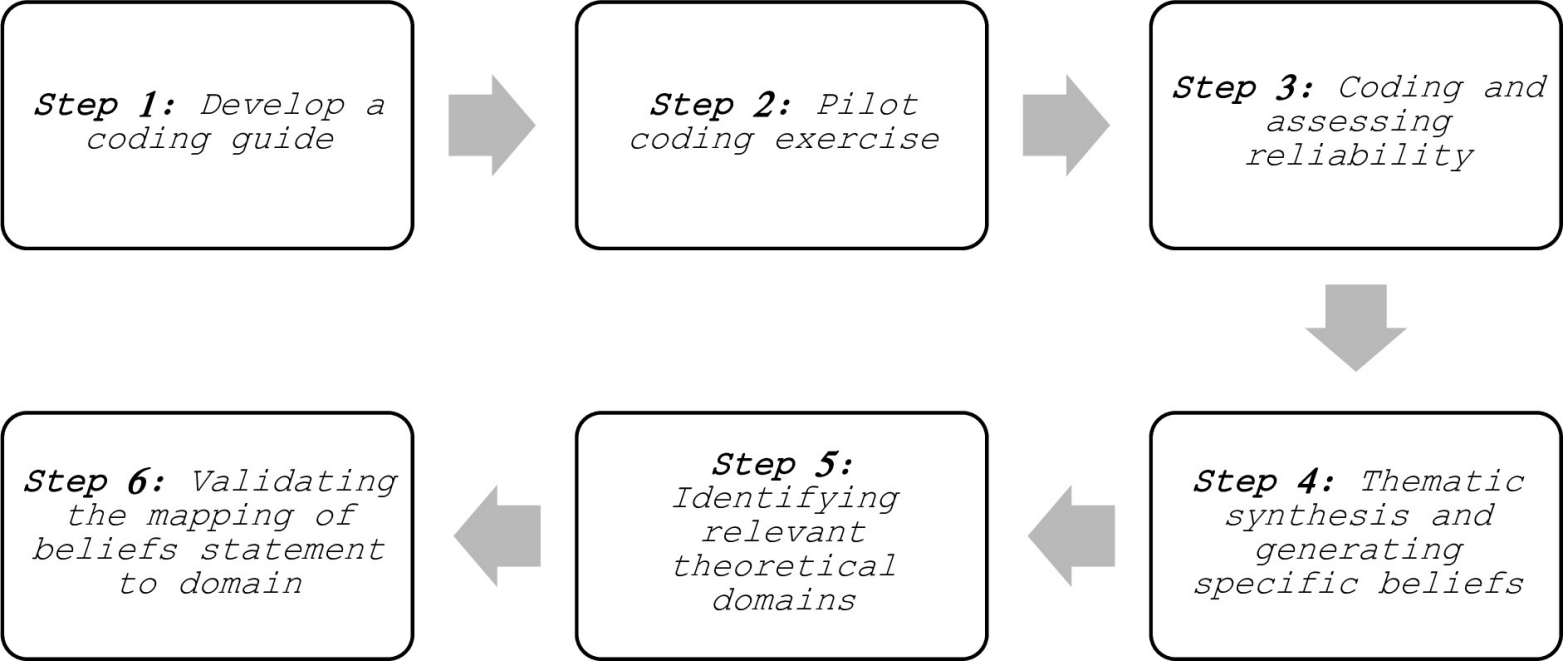
The data analysis process.

Data was imported and managed using QSR International’s NVivo 11 qualitative data analysis software [42]. The TDF framework [33] was used to explore the factors influencing whether service users were informed, involved and influential in the risk assessment and management. Both the interview responses from the lived experience questions and hypothetical questions were mapped to the domains of the TDF. Data analysis drew on established analysis methods used in previous literature [32, 43–45] and involved the following six steps (Fig 1):

*Step 1*: *Develop a coding guide*

A coding guideline was developed based on the 14 domains and 84 constructs from Cane, O’Connor and Michie [33]. To provide guidance and confidence that a piece of text represents a domain, statements of how the domain applies to the research context were also included in the coding guideline.

*Step 2*: *Pilot coding exercise*

To practice applying the TDF and to formulate a coding strategy, NA jointly coded two randomly selected interview transcripts with another researcher (LR) using the draft coding guide. The coding guideline was refined, and coding disagreements were discussed. A TDF expert (KM) also coded a transcript to enhance the accuracy of the final coding guide. A final version of the coding guide was agreed upon, which can be found in S4 Appendix.

*Step 3*: *Coding and assessing reliability*

NA coded the remaining interview transcripts independently. Participants’ responses within each transcript were coded into the relevant theoretical domain using the coding guideline. For example, *“But I think my mum may be the best one to talk about my risk…She would see things I don’t see”* was coded to the ’social influences’ domain. If a participant’s response represented more than one TDF domain, the text was coded to two or more domains. For example, *"I think it’s very important because… people in the service obviously need to know what risks that I am most likely going to be going through*, *because they don’t want me to go through it again"* was coded to both "reinforcement" and "beliefs about consequence". The second researcher (LR) coded the findings into the TDF domains for 20% of the transcripts (n = 2). Inter-coder reliability was assessed by calculating the percentage agreement/disagreement to measure consistency in coding within and across domains [46, 47]. Based on the TDF guide, reliability between two coders is considered acceptable if percentage agreement > 60% is achieved [32].

*Step 4*: *Thematic synthesis and generating specific beliefs*

NA generated statements representing the specific underlying belief for each participant’s response within each theoretical domain. A belief statement is defined as *’a collection of responses with a similar underlying belief that suggest a problem and/or influence of the beliefs on the target implementation problem’* [32 p.12]. Therefore, responses with similar underlying themes were grouped, and a summary belief statement was generated. For example, findings that suggest involvement could have ’prevented risk’, ’reduced risk’ or ’helped the individual feel safe’ were categorised as *"being involved in my risk assessment and management may have helped me to reduce my risks and improve my safety"*. New belief statements were created for responses that could not be grouped. A frequency count for each belief, capturing the number of participants who mentioned the specific belief in their response, was calculated.

*Step 5*: *Identifying relevant theoretical domains*

In line with previous publications [43, 44], the TDF domains relevant to the target behaviours were identified. Domains were identified as relevant if they contained a specific belief (step 4 above) that might be a potential barrier or enabler to the SDM component in risk assessment and management. In addition, three factors were considered when identifying the key domains: frequency of belief across interviews, presence of conflicting beliefs, and evidence of strong beliefs that might impact the behaviour. All these factors were considered simultaneously to establish the relevance of the domain in influencing the target behaviour.

*Step 6*: *Validating the mapping of beliefs statement to domain*

To ensure belief statements accurately represented the domain they had been generated within, a researcher with extensive experience using the TDF (KM) validated each belief statement. Blinded to the domain in which the belief statement had been generated within, KM was asked to assign a TDF domain to each belief statement. The agreement between the two researchers (KM and NA) was calculated through the number of items on which the two coders agreed divided by the total number of items, multiplied by 100.

## Results

### Participant characteristics

A total of 332 postal invitations were sent out to service users. Nineteen service users (6%) responded to the invitation and were screened for participation. Of these, six were excluded for the following reasons: insufficient English (n = 3), refused participation (n = 3). The remaining 13 service users (4%) took part in an interview. Participant characteristics are reported in Table 2.

**Table 2:** Service user participants’ characteristics.

	Service users (n = 13)
**Age**^1^, mean (standard deviation) and range	37 (12) 21–62
**Gender, n (%)**	
Female	7 (54)
Male	6 (46)
**Ethnicity, n (%)**	
White–UK or Irish	1 (8)
Mixed race	1 (8)
Bangladeshi	7 (54)
Asian–other	1 (8)
Black African	1 (8)
Black Caribbean	1 (8)
Black–other	1 (8)
**Mental health diagnosis, n (%)**	
Psychosis/Schizophrenia/Bipolar Disorder	12 (92)
Depression/Anxiety	1 (8)
**Daytime activity, n (%)**	
Unemployed	11 (85)
Voluntary work	2 (15)
**Time in mental health services, n (%)**	
10+ years	6 (46)
4–6 years	2 (15)
1–3 years	3 (23)
<1 year	2 (15)
**Length of treatment from current community mental health service, n (%)**	
10+ years	5 (38)
4–6 years	2 (15)
1–3 years	5 (38)
<1 year	1 (8)
**Relationship status, n (%)** ^2^	
Single	6 (46)
In established relationship	6 (46)
**Frequency of contact with carer, n (%)**	
Daily	7 (54)
Fortnightly	1 (8)
Weekly	4 (31)
Monthly	1 (8)

Missing Data: ^1^Age (N = 2);

^2^Relationship status (N = 1).

*All values represent n (%) or mean (standard deviation) and range

Most participants were female (n = 7, 54%) and from Black, Asian and Minority Ethnic (BAME) groups (n = 12, 92%). Nearly all the participants (n = 12, 92%) selected ‘Psychosis/Schizophrenia/Bi-polar disorder’ for their diagnosis. Most participants were recruited from the CMHT setting (n = 10, 77%) compared to EIS (n = 3, 23%). Length of contact with mental health services was most commonly 10+ years (n = 6, 46%).

### Involvement in shared decision-making in risk assessment and management

Prior to the TDF informed questions, participants answered questions about the assessment and management of risk. Ten service users (78%) said that no one, in their present CMHT/EIS, had carried out a risk assessment with them, developed a risk management plan with them or provided them with a copy of these documents. Therefore, they were asked questions exploring their views about not being involved, if they would have liked to be involved and the factors that may have influenced them from being involved.

In comparison, three participants (23%) answered yes to being involved in the risk assessment and management processes and thus were asked the TDF-based questions exploring their lived experience of barriers and enablers to them being informed, involved and influential in these processes. Findings were analysed separately but were both mapped to the TDF [33].

### Barriers and enablers to SDM in risk assessment and management

#### Theoretical domains

Thirteen theoretical domains (all TDF domains except Memory, attention and decision processes) were identified in findings from the service users’ interviews. Nine domains were identified in both sets of interview questions: hypothetical and lived experience questions. *Environmental context and resources* and *Optimism* were only identified in the findings from the lived experience questions, and *Beliefs about capabilities* and *Behavioural regulation* were only coded from responses to the hypothetical questions. All 13 participants mentioned *Knowledge* and *Social Influences*. Twelve participants mentioned *Intention*. Ten mentioned *Skills* and *Beliefs about consequences*. The domains with the most quotes were *Knowledge* and *Social influences*. *Environmental context and resources* and *Optimism* were mentioned by the fewest number of participants and supported by the least number of quotes.

### Belief statements

For the hypothetical questions, a total of 24 belief statements were created across 11 domains, ranging between 1 and 6 per domain (mean = 2.5, SD = 1.8). Table 3 details 24 belief statements, the frequency with which they were coded, and example anonymised quotations taken directly from the transcripts.

**Table 3:** Belief statements and sample quotes for responses to the hypothetical questions (n = 10).

Domain	Specific belief	*Sample quote*	No. of participants	Total no. of quotes
**Knowledge**	I am not aware of the risk assessment and management process	*“Has anyone ever discussed risk of safety with you*?*”*	**10**	**35**
		*“Not really*. *I can’t remember [risk] being discussed…They have gone through recognising my behaviour*. *That’s one thing with the psychologist*, *but not really…aware of risks and symptoms*, *those things*.*”*		
		*“As far as risk is concerned*, *the only kind of communication that I’ve had is where my psychologist has said*, *OK I’m not worried about you anymore*, *in the sense that there may have been a period where they thought…we’ve got to keep an eye on her and then he’s said*, *I’m not worried about you anymore”*		
	I do not know what is included in my risk assessment	*“Has anyone ever carried out a risk assessment with you*?*"*	**9**	**14**
		*“No*. *I think that it has been done in that it’s been done but I haven’t*, *no one’s said this is what we’re worried about…”*		
	I do not know what is included in my risk management plan	*“Has anyone developed a risk management plan with you*?	**9**	**15**
		*“No because I actually worked that out for myself and I explained to my Care Coordinator that at any time*, *if anything like this ever happened again*, *that I know that I can go see my oldest friend or speak with her or speak with my family*.*”*		
**Skills**	Talking about my risks and safety would be difficult	*“it would be a challenge to share my experience”*	**5**	**6**
		*“Things that I don’t know that I’m at risk of and I don’t even know…that would be difficult”*		
		*“Wanting to discuss it in the first place*, *I think*. *A lot of it makes me really uncomfortable but mental health is an uncomfortable subject to talk about*. *Yeah just having to remember that I did those things*. *I’ve also self-harmed and it’s just uncomfortable because when I seem to be OK I’m not in the depths of depression*, *I’m not psychotic*, *I’m kind of like I am now*. *Just to think back and remember that I used to cut my arms to ribbons or that I used to strangle myself it’s hard to*, *I’d just rather forget about it but I know I can’t*, *I know I can’t so”*		
	Talking about my risks and safety would be easy	*“Easy*, *easy*.*”*	**5**	**6**
**Social/professional role and identity**	My risks are low, so I do not need to be involved in my risk assessment and management plan	*“Because there’s not really much to discuss and from the list you went through*, *I had isolation but about the self-harming*, *nothing…”*	**5**	**11**
**Beliefs about capabilities**	I would feel confident being involved in my risk assessment and management plan	*“Very confident actually*, *I think I’ll be able to give my own input in it as well*, *and just be able to take it away as well*, *it’s something to take away*, *good information”*	**6**	**7**
	I would not feel confident being involved in my risk assessment and management plan	*“No*, *I wouldn’t be confident*.*”*	**1**	**1**
**Beliefs about consequences**	I would have been able to better understand and manage my risks if I was involved	*“The benefits would be maybe a greater understanding*, *and also recognising your symptoms and risks that you have*, *and other benefits*.*”*	**6**	**13**
		*“It would have helped me to understood what it was that is worrying for the team*. *I know the things that I worry about but I think I would’ve like to have known what it is that’s concerning them*. *What they think is a risk*.*”*		
	I think having more involvement in my risk assessment and management plan would help me keep well	*“It would be a very good benefit because I tell the story of what happened*, *and when they hear my story they might do something about me*, *they might help me to be well…they might help me to feel safe…”*	**5**	**6**
		*“I think a number of things like medication*, *talking it through*, *treatments*, *talking therapy*, *different variety of things would be quite helpful in terms of keeping yourself well*, *and also what you can do outside*, *more activities*. *I’ve noticed I was really depressed at home*, *and I was just sitting at home doing nothing*, *and that really depressed me…”*		
	Being involved in my risk assessment and management plan may have helped me to reduce my risks and improve my safety	*“I think I would have known the fact that*, *things like going out*, *walking around with scissors is a risk it’s*, *that’s not something I should be doing so I’d be aware of it*, *of what the risk is and work towards reducing that risk*.*”*	**4**	**5**
	Being involved in my risk assessment and management plan would keep me safe	*“That will keep me in a line*, *in a boundary*, *where I’m safe*.*”*	**3**	**4**
	I would feel stigmatised and labelled if I had seen past incidents recorded on my risk assessment	*“I wouldn’t be too pleased but I would understand why it had to be on there*. *But there is no time limit to these things though is there*? *If it happened when you‘re 16 and now you’re 47 it would still be on your risk assessment wouldn’t it*? *So*, *I mean I guess I wouldn’t mind but it wouldn’t be pretty to look at*, *but then again”*	**1**	**4**
**Reinforcements**	My openness and honesty would have enabled me to be involved in my risk assessment and management plan	*“Because I’m more open*, *isn’t it*? *So*, *I don’t like to hide*, *tuck away things behind closed doors and leave it*. *I like to deal with the problem at the time*.*”*	**5**	**6**
		*“Just being involved and not people making decisions without me knowing*, *you know*? *Without me knowing or writing or talking about me and I have no idea that’s going on so yeah I would*, *and I don’t want any wrong information to end up you know like on my mum’s medical record it says she’s allergic to penicillin*, *she isn’t*.*”*		
	I feel it is important that I am involved in my risk assessment and management plan	*“I would say they should sit and talk to you before you get worse”*	**3**	**7**
**Intention**	I would have been willing to be involved in my risk assessment and management plan	*“I wouldn’t have had any problems or any issues with it at all*. *If she turned around and said look…because this is what we have to do*, *this is the process*, *the procedure*, *I’d be more than happy to go through it*.*”*	**9**	**18**
		*“…Yeah and I think it would be good to review it*, *to review the risk assessment*, *I don’t know maybe like every quarter or something*?*”*		
**Goals**	I feel it is important that I have a say in my risk assessment and management plan	***“****Really putting my own individual stamp on it*. *So I could turn around and tell this is my risk assessment which I have in my head and again connecting with it in my head is risk management*.*”*	**5**	**11**
		*“I think maybe a bit apprehensive maybe*, *I’m not the most confident of people but I would have liked to have been involved as this is me*, *it’s me they’re talking about*, *an actual person and not a statistic so*.*”*		
		*“I would like a say in*, *in what they*, *what*, *what they saying*.*”*		
**Social influences**	I think my friends and family would support me to be involved in my risk assessment and management plan	*“But I think my mum may be the best one really to talk about my risk…She would see things I don’t see*. *She’d see things I do that I probably wouldn’t recognise that I do…”*	**9**	**21**
		*“I think all the people in terms of your care and your family*, *maybe one member*, *close member of the family should be involved in the risk assessment as well*.*”*		
	We need better communication between service users and professionals about risk	*“In the future*, *communicate*, *basically*. *Communicate and have goals*, *probably*. *Goals in how you can take action for certain*, *you know*, *different types of risk that’s imposed at yourself*, *and ways that you can deal with them as well*.*”*	**7**	**14**
	Professionals’ make all the decisions about risk assessment and management planning	*“I know that from a professional point*, *they can’t tell me everything that they’re discussing because I’m the patient and some of it’s not relevant but just*, *if we had a meeting before*, *this would be good*.*”*	**2**	**3**
		*“Nothing as I like to be involved anyway yeah*, *I like to be involved anyway*, *I like to be cooperative*, *I find people treat you better when you’re cooperative*. *You’re less likely to fall back into a psychiatric unit if you’re cooperative so*.*”*		
	If the professional and I disagreed about my risks, it would make it difficult to be involved in my risk assessment and management plan	*“You can be a bit defensive*, *that can be quite challenging*, *no I don’t*, *not like that*, *or I won’t be like that*, *so you might be in denial”*	**1**	**1**
	There needs to be more carer involvement in risk assessment and management planning	*“I would make it more open*, *and I would get other*, *I think I wouldn’t just be me involved it would have to be my family as well*. *But I know not a lot of people*, *there are a lot of people who don’t get on with their families but I think the family involvement and the friends’ involvement is very important because like I said they can see things that you do that they would deem a risk that you just think are normal anyway*.*”*	**1**	**1**
**Emotions**	I believe I have not been involved in my risk assessment and management plan because my mental health has been good	*“I don’t know*, *maybe they think that I’m OK maybe”*	**4**	**8**
		*“Probably because the way I come across* …*I’m very cooperative*, *I always attend my appointments*, *I always take my medication*, *I engage in*, *with the doctors and with my care worker and I always talk about things*. *I kind of*, *I don’t always share everything but as much as I can and I kind of just like to look like I’m interested*. *So yeah I think*, *I think maybe the way I present myself and the way I come across*. *I’ve not had a psychotic episode in years now”*		
	Talking about my risks would make me feel distressed and/or upset	*“The only thing that I did think of is because I tend to over analyse*, *the team may have been worried if they tell me that*, *we’re worried that you might do this or that you’re going to harm yourself or others*. *Because*, *I don’t know…If it was because they thought that would just add to my worry and anxiety but I think it would’ve helped because then I know what it is that’s concerning them*.*”*	**3**	**7**
		*“…it’s a bit emotional to go through as well*, *and to talk about it makes you feel more thinking and everything*, *and it could be* …*quite upsetting*		
**Behavioural regulations**	A risk tool might help me to be more involved in risk assessment and management	*“I think something to do with communication maybe*. *Messaging*, *maybe text messaging or like an app or something you know…”*	**2**	**2**

For the lived-experience questions, 21 belief statements were created across 11 domains, ranging between 1 and 5 per domain (mean = 1.9, SD = 1.3). Table 4 details 21 belief statements, the frequency with which they were coded, and example anonymised quotations taken directly from the transcripts.

**Table 4:** Belief statements and sample quotes for responses to the lived-experience questions (n = 3).

Domain	Specific belief	*Sample quotes*	No. of participants	Total no. of quotes
**Knowledge**	I know what is included in my risk assessment and management plan	*“It included when I’m going to have a breakdown*, *that I’m going to be praying*, *that I’m going to lose sleep*, *they*, *she basically handed out a few cards and stuff and it’s I’ll lose my sleep*, *I’ll pray more*, *I’ll*, *the television start talking to me and I’ll pace about*, *I’ll lose my appetite*, *I’ll lose weight*, *that’s the*, *that’s when I’m getting unwell*.*”*	**3**	**20**
		*“The risk management is on a regular taking medication*, *on a regular seeing my doctor*, *and telling her what I’m experiencing”*		
		*“Yeah*, *a lot of people did like I think I met a couple of doctors about it and they said I could relapse at some point…they didn’t really say how depending when it is*, *but they did talk about the risk part*. *I mean like do I have any thoughts and stuff like that about hurting someone or hurting myself*, *and obviously I don’t think like that*, *hurting other people*.*”*		
	Being aware of my risks makes it easier for me to be involved in my risk assessment and management plan	*“And I think that really helped really in a good way that that’s what would make me talk about the risk stuff…because talking is really easy for me about how I experienced and feeling about this condition*.*”*	**3**	**5**
**Skills**	Talking about risk with professionals is difficult	*“it would be a little bit hard obviously going through the risk*, *but I would say I’ll be*, *I will still be positive to talk about what happened and stuff like that*.*”*	**2**	**3**
**Social/professional role and identity**	My mental health team make all the decisions about my risk assessment and management	*“Then again they seemed to make the decisions for themselves*, *they don’t really ask for your opinion or whatever*.*”*	**2**	**8**
		*“I guess you have to be controlling but you need to know my opinion as well*, *you need to ask for my views as well*. *Sometimes*, *I don’t know what to say because they’re prof*, *they got degrees*, *master degrees whatever and just because I feel stressed for the moment and then you increase my medication I don’t understand that”*		
	Professionals are responsible for my risk assessment and management	*“I prefer the mental health know what they need to do when a patient is unwell because people committing suicide I still think it’s partly the mental health’s fault*. *They need to take action more sooner*, *there’ll be a lot more less committing suicide*, *so they need to know what they need to do the psychiatrists*, *the occupational therapists*, *the psychologist*, *the whole treatment team*.*”*	**1**	**4**
**Optimism**	I am optimistic that I will be involved in future risk assessment and management planning	*“How optimistic are you that in the future you will be involved in identifying or managing your risks*?*”*	**2**	**3**
		*“Positive*.*”*		
		*“I would like to be but I don’t know it’s just there’s a control there…”*		
**Beliefs about consequences**	Being involved in my risk assessment and/or risk management has helped me to reduce my risks and improve my safety	*“I mean the benefits are that I know what the risks are and I know how to improve on them and I know how to do it by talking to the psychologist or anyone in this service*, *because it just helps improve what risks I don’t have to have*, *which just makes it better because I’m talking it through with a person*, *which sort of helps*.*”*	**2**	**5**
**Reinforcement**	My openness and honesty enabled me to be involved in my risk assessment and management	*“And I think this stuff is really good for me in case*, *because I don’t know if I’m at risk at the moment but I feel going to these meetings and stuff and talking to them*, *sometimes they even come to my house to talk*. *[care coordinator] does*, *he comes to my house and we just have a chat and stuff*, *how I’m doing*, *how I’m feeling and stuff like that*. *So this stuff actually does help*.*”*	**2**	**9**
		*“Being honest is a good idea but it’s not always a good idea in the sense that you’ve got to use your head a little bit*, *you’ve just got to say*, *OK I developed it*, *rather than saying*, *someone put a curse on me because they*, *in this country or the psychiatrists in this country just what they want you to say*, *yes you got it*, *rather than saying someone put a curse on me*, *you know*, *but they want you to say that so you’ve got to say that”*		
	The opportunity to better understand and manage my risks encourages me to be involved in my risk assessment and management planning	*“I feel like talking it out can help and understand how you are feeling and stuff*, *and it can help you improve on certain things which made you unwell in the first place*. *So*, *I would say it’s a good thing for other people to get involved*.*”*	**2**	**7**
**Intention**	I intend to be involved in my risk assessment and management planning in the future	*I think I would discuss it because I’m a changed person*, *I’m not as*, *as I was before* …*”*	**3**	**3**
**Goals**	Keeping myself well encourages me to be involved in my risk assessment and management	*“It does help*, *it does help and sometimes it’s not always in my control*, *it’s*, *schizophrenia when I’m not well I’m not always in control*. *I can be in control to a certain extent*, *but I’m not always in control and I need to go into the crisis house or if I’m having a fully blown breakdown to the hospital and to be closely monitored…”*	**3**	**9**
		*“What goes through your mind in the appointments you have with your doctor*, *and she’s discussing risk*?		
		*“Recovery*. *Because I want to be well*.*”*		
	I feel it is important that I am involved in my risk assessment and management	*“I think it’s very important because obviously*, *people in the service obviously need to know what risks that I am most likely going to be going through*, *because they don’t want me to go through it again*. *They want to help me improve or things like help me go*, *help me be better than I was before*.*”*	**3**	**8**
	I want to have a say in my risk assessment and management plan	*“And I think it’s a good idea so I could come in and talk to someone on a daily basis about my mood*, *my risk and stuff like that*, *which I could help improve on it because I think talking is a good thing with professionals who know how to deal with this situation*.*”*	**3**	**5**
**Environmental context and resources**	Meeting with my mental health professional more often would make it easier to be involved in my risk assessment and management plan	*“Yeah*, *I think it was very helpful that it was more because usually it’s just every month or so*, *and I could see the consultant every two weeks and we would talk about how I’m feeling and stuff and how great my mood and everything*. *From numbers mostly and on a paper*, *and how I’m feeling from the two weeks before*, *how I’m feeling now and stuff like that*. *And I think that really helped really in a good way that that’s what would make me talk about the risk stuff”*	**2**	**2**
		*“More sessions*.*”*		
**Social influences**	My mental health team help me to be involved in my risk assessment and management planning	*“Maybe the psychologist*, *I think I was doing some risk things like what I could do if at a certain stage like maybe going back to hospital*, *if I was unwell again*. *I mean at a point where it’s to the extreme*, *where I’m really unwell*. *It was with one of the psychologists*, *we talked about the things that I did and stuff*, *and what can I do to interact with them to make it better*.*”*	**3**	**17**
	There needs to be better communication between service users and professionals about risk	*“I’d probably just talk to my care co-ordinator about it*, *so maybe I could learn more about other risks as well*. *Or*, *the psychologist could also help*. *I could*, *I have talked about it like through my care co-ordinator that I want to see the psychologist again which could help me just talk things out on a daily basis about it*, *so*.*”*	**2**	**3**
	If the professional and I disagree about my risks, I find it difficult to be involved in my risk assessment and management planning	*“It’s difficult sometimes*, *when I say to my psychiatrist that I’ve feeling aggressive or whatever she asks me a question*, *do you feel like harming anyone and I say*, *yes I do*, *but the thing is that it’s not that I want to do it it’s just a thought I guess and it’s not that I want to do it*, *you know*. *They need to be more professional about that as well”*	**2**	**3**
		*“…other people*, *they think that going outside is a risk*, *when*, *while I feel going out is an opportunity*.*”*		
	My friends and family help me to be involved in my risk assessment and management planning	*“I think my family are very important in it*.*”*	**2**	**3**
	I prefer to talk about risk with professionals on a one to one basis	*“Social*, *socially*, *socially*. *I’m not very good socially*, *so*, *when there’s a lot of people talking about it*, *I’m not very good*, *but when it’s one to one I’m very good with it*.*”*	**1**	**1**
**Emotions**	If my mental health is poor, I find it difficult to be involved in my risk assessment and management planning	*“… it’s a little bit easy but sometimes it is hard because some of the risks you don’t really know*, *because some actions when you do you don’t really know what*, *remember what you’re doing*, *so it’s like sometimes you just do things without actions*, *without knowing what you’re doing*. *So some of the risks you don’t really know what they are unless the doctor*, *the consultant tells you what you’ve done and stuff*.*”*	**3**	**14**
		*“Because if I became very unwell*, *I won’t be responding*, *and the risks*, *I’ll be a risk to myself*, *I’d be a risk to myself*.*”*		
	I feel good about being involved in my risk assessment and management planning	*“I feel good about it because I know the risks”*	**1**	**2**

### Inter-rater reliability

Inter-rater agreement was calculated for 20% of transcripts across the 14 TDF domains and ranged between 85%-100%. When blinded, a TDF expert was asked to map the belief statements onto a domain. For the hypothetical questions, 13 (54%) of the 24 beliefs were mapped by the expert onto the intended domain, whereas 11 (45%) beliefs were mapped onto a different domain. After discussion between the expert (KM) and the first author (NA), it was agreed that 16 (67%) beliefs should remain mapped to the intended domain, whereas 8 (33%) beliefs were re-mapped based on the expert’s recommendation. For the TDF based questions, 15 (71%) of the 21 beliefs were mapped onto the intended domain, and for 6 (29%) beliefs, there was disagreement. The two researchers met to discuss and reach a consensus. After discussion, they agreed that 16 (76%) beliefs should remain mapped to the intended domain. Five (24%), however, were re-mapped to the domain identified by the expert.

### Beliefs mapped to the TDF domains

In the following section, the key beliefs within each of the domains of the TDF are summarised. A definition is provided when introducing each domain, this is based on Cane, O’Connor and Michie [33].

#### 1. Knowledge

Knowledge is defined as *“an awareness of the existence of something”*.

Most of the sample (n = 10, 78%) said they were unaware of the risk assessment and management process. They could not recall professionals discussing risk with them or involving them in developing their assessment or plans. Three service users (23%) reported being aware of the process and professionals within the CMHT/EIS discussing risk with them. Two participants recalled receiving information about their risk of violence to others and professionals, mainly their psychiatrist or psychologist, regularly reviewing risk with them. All three said that they had received advice from professionals about recognising their relapse triggers and were provided with a copy of their risk assessment and management plan.

#### 2. Skills

Skills is defined as *“an ability or proficiency acquired through practice”*.

Half of the service users who answered the hypothetical questions said they would have found it easy to discuss risk with professionals. However, others believed sharing their experiences with risk would be a challenging task. For example, a service user said discussing their risk of suicide could have heightened their thoughts of taking their life, and another believed discussing incidents that occurred when they were unwell would be uncomfortable. Service users who answered the lived experience questions considered risk a personal topic challenging to discuss with professionals when they felt judged. Nonetheless, some individuals said they were still optimistic about discussing risks with professionals despite these discomforts and challenges.

#### 3. Social, professional role and identity

Social, professional role and identity is defined as *“a coherent set of behaviours and displayed personal qualities of an individual in a social or work setting”*.

Service users who reported not being involved believed that this may be because they considered themselves to be low risk, or because they had been ’cooperating’ and engaging with services and treatment.

Service users who answered the lived experience questions spoke about professionals making all the decisions about their risk assessment and management. There was a sense of powerlessness in some reports with participants mentioning professionals’ qualifications and titles, feeling controlled and unheard. For example, a service user spoke about their opinion not being considered in key decision-making about changes to their medication. Another perceived risk management as the professionals’ responsibility and believed that some risk could be prevented if professionals had acted faster.

#### 4. Beliefs about capabilities

Beliefs about capabilities is defined as *“acceptance of the truth*, *reality*, *or validity about an ability*, *talent*, *or facility that a person can put to constructive use”*.

Most service users who had not been involved believed that they would have felt confident in identifying and managing their risks. One service user elaborated and said that they would have valued the opportunity to contribute and learn about their risk factors. Another service user, however, believed that they would not have felt confident identifying and managing their risks.

#### 5. Optimism

Optimism is defined as *“the confidence that things will happen for the best or that desired goals will be attained”*.

One service user who answered the lived experience interview questions reported feeling optimistic that they would be involved in identifying and managing risk in the future. Another participant said they would like to be involved, but this depended on professionals whom they perceived as ‘in control’.

#### 6. Beliefs about consequences

Beliefs about consequence is defined as *“acceptance of the truth*, *reality*, *or validity about outcomes of a behaviour in a given situation”*.

Service users who reported no involvement said that talking about their risks could have helped to prevent them from happening. They spoke about the benefits of understanding their risk, the consequences of their risks, and sharing their story. Participants believed that involvement could have improved their safety and helped them better understand how to manage their risks.

Risk was described as a sensitive and emotive topic, and one participant spoke about potentially feeling stigmatised and labelled by seeing something that had occurred in the past remain on their record. In response to the question, “Why do you think you have not been involved in your risk assessment?” a service user believed professionals might have been worried about causing them distress, which they agreed with, as they too believed that a conversation about risk could have heightened their thoughts of harming themselves.

Some service users believed that professionals missed an opportunity to support them in understanding and managing their risks. For instance, a service user reported having trouble separating their distorted thoughts from reality and felt that if professionals had explained the risk (and challenged their thinking), this could have supported them in keeping well.

Service users who answered the lived experience questions believed that being involved increased their awareness of their risks and made them more equipped to minimise and manage them.

#### 7. Reinforcement

Reinforcement is defined as *“increasing the probability of a response by arranging a dependent relationship*, *or contingency*, *between the response and a given stimulus”*.

A couple of participants who answered the lived experience questions said that the opportunity to better understand and manage their risks encouraged them to be involved in their risk assessment and management planning. In addition, they believed that their openness and honesty enabled them to be involved. They spoke about attending meetings and being willing to talk to professionals about their feelings, which helped manage risk. One participant spoke about being honest but also being aware of how professionals could interpret their beliefs, e.g., a curse being put on them, and thus adjusting how they spoke to professionals about these risks.

Service users who had not been involved also believed their openness and honesty may have encouraged them to discuss risk with professionals. They said they would have liked to have been involved in decision-making about them and had the opportunity to review the accuracy of information on their record.

#### 8. Intention

Intention refers to *“a conscious decision to perform a behaviour or a resolve to act in a certain way”*.

Most participants who answered the hypothetical questions said that they would have been willing to be involved in their risk assessment and management planning. They believed that they would have engaged or ‘cooperated’ in discussions about risk and would have liked to review the risk assessment documents. One participant perceived themselves as low risk, so did not feel it would have been beneficial for them to be involved in these processes.

The service users who answered the lived experience questions said that they intended to continue to be involved in their risk assessment and management planning in the future.

#### 9. Goals

Goals refers to *“mental representations of outcomes or end states that an individual wants to achieve”*.

Service users who answered the hypothetical questions discussed the importance of having a say and sharing their story. For example, a service user emphasised that the risk assessment and management were ’about them’ and thus, professionals should have involved them and allowed them to contribute to decision-making.

The opportunity to keep well, reduce risk and be able to contribute to the process motivated service users who answered the lived experience questions to be involved. However, one service user talked about being prescribed medication instead of being listened to when he reported feeling stressed. Consequently, the service user thought it best not to be open with professionals.

#### 10. Environmental context and resources

Environmental Context and Resources is defined as *“any circumstance of a person’s situation or environment that discourages or encourages the development of skills and abilities*, *independence*, *social competence*, *and adaptive behaviour”*.

Two service users who answered the lived experience questions believed that more sessions with professionals, such as a psychologist, could have enabled them to be more involved in discussions about their risks.

#### 11. Social influences

Social influences refer to *“those interpersonal processes that can cause individuals to change their thoughts*, *feelings*, *or behaviours”*.

Nearly all the service users who answered the hypothetical questions believed that their friends and family would have supported them in being involved in risk assessment and management planning. Service users identified close family members such as their mother, father, siblings or partner as people who should be involved. Participants believed that professionals should have involved their family or carers for several reasons, including to help them keep safe, understand their risks, provide their carer with knowledge about their risks, and contribute to the process. The importance of family involvement was stressed by a participant who believed their family might have recognised risks they might not have.

In addition, participants believed disagreements could be a potential barrier to discussing risks with professionals. For example, a service user admitted that if professionals had involved them in their risk assessment, they might have acted defensively or been in denial about their risks. Two participant responses indicated power dynamics between them and professionals in decision-making. For example, a service user accepted that professionals could not tell them everything about their risks because they were the ’patient’ but said they would have found it helpful to hear the positives about their risks, e.g., how far they had progressed. Another service user suggested that being ’cooperative’ might be why professionals decided not to involve them in their risk assessment and management plans. They said they ’cooperated’ because it made professionals treat them better and they were less likely to end up in hospital.

Participants who answered the lived experience questions about their involvement also believed that their friends and family helped them to be involved, as they understood their condition well and had their best interest. They named several professionals who enabled their involvement in the risk assessment and management processes, including their psychiatrist, psychologist, care coordinator, and support worker. They were confident that these professionals had discussed their risks, relapse triggers and crisis plans with them. Generally, they described these conversations as helpful and supportive in keeping well. One service user, however, described professionals as in control of decision-making. They believed that professionals made all the decisions and did not ask for their input. A couple of participants spoke about being unable to talk openly with professionals about their risks or what they believed happened to them as they feared the consequences. One service user said they preferred to discuss risk on a one-to-one basis.

Service users who answered both the hypothetical and lived experience questions suggested that better communication could help to increase their involvement in the risk assessment and management process. Those that had not been involved talked about professionals detecting their risks early, sitting down and talking to them, being honest with them and explaining the consequences of their risky behaviours to them.

#### 12. Emotions

Emotions is defined as *“a complex reaction pattern*, *involving experiential*, *behavioural*, *and physiological elements*, *by which the individual attempts to deal with a personally significant matter or event”*.

Service users who answered the hypothetical questions suggested that professionals might not have involved them because their mental health had been good or because they understood their relapse triggers and crisis plan. They remembered professionals informing them that they were no longer concerned about them, but they could not recall having been involved in a discussion about their risks. Some recognised that discussing their past risk could cause them distress or upset and that this may have been a barrier to their involvement.

The service users who answered the lived experience questions also talked about their mental health impacting their level of involvement in risk assessment and management planning. They described professionals discussing their relapse triggers and crisis plans with them when their mental health was good, but also recognised the difficulty in talking about risk when they lacked awareness of the behaviours and incidences that had occurred when they were unwell. They described professionals needing to remind them of their past behaviours and admitted that if their mental health was poor, they probably would not have responded well to discussions about their risks with professionals.

#### 13. Behavioural regulations

Behavioural regulation is defined as *“anything aimed at managing or changing objectively observed or measured actions”*.

A couple of the service users who answered the hypothetical questions proposed a risk tool to help increase their involvement in the risk assessment and management processes. Examples that they provided included a software application, text messaging service or leaflet.

## Discussion

Through the use of the Theoretical Domain Framework (34), this qualitative interview study explored service users perceived barriers and enablers to SDM in risk assessment and risk management.

Most service users in this study reported a lack of awareness regarding the contents of the risk assessment and management documents, as well as minimal involvement in these processes. Among the few service users who reported some level of involvement, they mentioned that professionals had advised them about their risks and included them in the development of the risk management plan. However, it was unclear the extent they actively participated in risk assessment and management as engaged partners in SDM. These findings are consistent with those of previous studies [4, 17, 27], which have found that service users are often not involved or aware of the content of their assessment or plan. It has been suggested that professionals not involving service users in risk assessment deny opportunities to understand safety concerns and the consequences of risk [4].

Despite most participants reporting a lack of involvement, we found shared perceptions of barriers and enablers in risk assessment and management, as most TDF domains (9 out of 13) were identified in both hypothetical and lived experience interview responses. Participants in both groups spoke about the difficulty in discussing sensitive topics with professionals (Skills) and identified mental state (Emotions) as a potential barrier to shared decision-making. These findings align with previous research on mental health professionals’ perspectives of shared decision-making and experience in working with inpatients who are suicidal [48, 49]. Participants also recognised the importance of enhancing safety (Beliefs about Consequences) and the support from family and friends (Social Influences) as key enablers, which is consistent with evidence highlighting the role of social support in achieving better mental health outcomes [50]. Differences between the groups may be attributed to having lived experience in the assessment and management of risk. For instance, the lived experience group noted the frequency of meetings (Environmental Context and Resources) as a barrier to involvement but expressed optimism in being involved in the future, while those who answered the hypothetical questions identified confidence (Beliefs about Capabilities) as a potential influencing factor.

Shared decision-making requires more than consulting with the service user but power-sharing in the decision-making process [23]. In the present study, however, participants described professionals as being in control and making all the decisions about their risks because they are the ’patient’. Studies have found that some service users position themselves as passive recipients in the risk assessment process [4], or perceive the assessment as a professionals’ task [28]. It has also been highlighted that not all service users want the responsibility of making their own healthcare decision [25, 51]. Historically, paternalism was the dominant approach used in decision making. Although this approach can still be justified in light of beneficence (to promote good) and non-maleficence (to prevent harm) [52], nowadays, in most healthcare situations, it is considered ethically inappropriate [53]. Mental healthcare is often considered an exception due to complexities with mental capacity and managing risk. Nonetheless, models of SDM highlight the importance of determining the service users preferred role in decision-making [54, 55]. It may be that some service users desire to receive information but prefer for the professional to lead on decision-making.

Some service users reported that they had not been involved in risk assessment and management because their mental health had been stable, and they had been compliant with professionals. They suggested that cooperating with professionals resulted in better treatment and reduced the likelihood of readmission to psychiatric hospital. These findings highlight a sense of powerlessness and acquiescence with professionals to avoid undesirable outcomes, and this coincides with the notion of “playing the game” described in forensic mental health studies [26]. Findings in forensic mental health studies indicate that some service users engage in risk management processes, albeit through a performance of compliance, and suppress their frustration and disagreement to achieve a greater level of autonomy and freedom [28, 56]. However, it is important to note that the current study was carried out in community mental health services. At the time of the interview, none of the service user participants were under any restriction. A possible explanation for service users positioning themselves as passive recipients might be to avoid coming in to contact with restrictions and the power imposition of psychiatry. Indeed, some service users reported a reluctance to talk openly with professionals about their risks due to fear of the consequences. Previous research has also found that fear of repercussions, such as losing services or receiving poorer care, hinder service users from raising safety concerns [57]. If service users do not feel comfortable discussing risk openly with professionals, services may not be aware of potential risk factors, which might reduce the effectiveness of the risk management plan.

Another key finding was that service user participants considered their family and friends pivotal in supporting them to be involved in the risk assessment and management processes. It has been reported elsewhere that carers too desire to be involved in these processes with the service user. For example, Clancy, Happell and Moxham [16] found that a lack of communication about risk from mental health services made it more difficult for carers to support family or friends and negatively impacted carers’ feelings of safety. In contrast, inclusive communication about risk increased carers’ confidence in professionals and made them feel that their loved one was well taken care of. Coffey et al.’s [4] findings indicated that carers had limited input to risk assessment and management planning to the extent that they felt unsupported and left to manage situations of risk themselves. Finally, a systematic review of carers’ experiences of involuntary admission under mental health legislation found that carers are often not provided with information to help them feel confident in caring for the individual, such as information about the patient’s illness, care and treatment planning, discharge planning and legal rights [58], therefore carers reported feeling ill-equipped and fearful about managing risk and protecting themselves. Together, these findings highlight the importance of involving carers in the risk assessment and management processes, as they can be instrumental in increasing the service user’s engagement and understanding, and they too require support in managing risk.

Most service user participants in the current study expressed a willingness to be involved in the assessment and management of risk but also recognised the challenges associated with discussing and remembering sensitive and emotive topics. These findings highlight the importance in considering the service users’ preferences, and readiness to engage in sensitive risk discussions. Hawton et al. [15] advocate for a shift in approach to assessing suicide that includes therapeutic risk assessment, formulation and management, and which is based on open dialogue, active listening and validation of feelings without judgement. The assessment of severe and sensitive risks, such as suicide, can be anxiety-provoking for professionals also [59]. Therefore, a co-delivered and co-produced training intervention by service users and mental health professionals could help facilitate sensitive and emotive discussion about risk. Grundy, Walker [60] evaluated a co-delivered training package for community mental health professionals focused on involving service users and carers in care planning. They found that professionals valued the co-production training model, and believed it improved their understanding and skills in engaging service users and carers in care planning discussions.

Potential benefits of involvement from service users’ perspectives included gaining a better understanding of risk issues and enhancing their ability to maintain well-being and safety. Mental health research advocating for collaborative safety planning or positive risk management strategies further underscore the potential benefits of implementing shared decision-making in these practices [61–63].

### Strengths and limitations

Research indicates that people from minority ethnic groups are under-represented in clinical and health research [64], and lived experience involvement in research may improve outcomes and research impact [65, 66]. This study’s design and methods were informed through discussion with service users and carers and included a high proportion of ethnic minority participants. The study identified a comprehensive list of the barriers and enablers to SDM in risk assessment and management using the TDF. A TDF expert helped develop the interview schedule and validate the belief statements generated from the study’s findings. Rigorous methods of data analysis were used to reduce bias, including double coding and reliability checks.

The study, however, was limited in several ways. First, implementing SDM in assessing and managing risk involves several behaviours, and the barriers and enablers may vary across these behaviours. Stacey, Felton [23] separated SDM into three components (i.e., informed, involved and influential), and our questions covered the three components but primarily focused on involvement. To explore this issue more comprehensively, it would have required repetition of the interview questions for each individual behaviour, which would likely have been tedious and lengthy for participants.

The qualitative nature of the interviews and sampling within one inner-city service provider precludes findings that are transferable to the wider population. The sample included service users of predominantly South Asian and Black African backgrounds, which reflects the high proportion of BAME residents in the borough where data was collected. Also, the low response rate (6%) may impact the representativeness of the findings. While qualitative research does not aim for statistical generalisability [67], the low response rate may limit the diversity of perspectives captured. However, the insights gained still provide valuable understanding of service users’ experiences of shared decision-making in risk assessment and management and may be transferrable to other services and settings. In addition, although risk assessment and management remain intrinsic activities in psychiatric practice and research in this area is still scarce, the interviews were conducted several years ago, and prior to the pandemic. However, it is highly likely the findings are still relevant to both clinical practice and research.

It is important to acknowledge that SDM should ideally include the carers’ input (if appropriate). As the present research only focused on the perspectives of service users, insight might have been limited by a lack of examination of carers’ experiences, such as in Jackson et al. [68] study of carers’ involvement in research assessment. Finally, the reported lack of involvement in risk assessment may be influenced by the length of time a person has been receiving mental health care. For instance, those who have been using community mental health service for many years might not recall their initial assessments. Additionally, service users with low risk levels might have had fewer or less intensive discussions about risk. Furthermore, even when risk is routinely assessed, it may not be described in overt terms to avoid causing discomfort or reinforcing stigma. Research has found that professionals adapt their language, framing discussions around "safety" rather than "risk" [16, 49], which may obscure participants’ memories of being actively engaged in risk discussions.

## Conclusion

The findings of this study show that service users generally support the use of shared decision-making in risk assessment and management, but in practice, this does not seem to be happening consistently or explicitly. Most service user participants reported having not been involved in identifying and/or managing risk and had often not seen their risk management plan. Perceived barriers included power dynamics between professionals and service users, difficulty talking about sensitive risk topics, and the emotional impact of engaging in these discussions. Recognising the importance of enhancing safety and greater involvement of family members or carers were largely seen as enablers of SDM in these processes. Some of the possible complexities of SDM in risk assessment and management for both service users and staff are explored. To our knowledge, this is the first study to have interviewed service users regarding what they perceive to be the barriers and enablers to SDM in risk assessment and management. Together with the findings from our review of mental health professionals’ experiences, these insights may be helpful to clinicians in enhancing their practices related to collaborative safety planning and/or can be used for targeting behaviour change in future intervention designs. However, further work is needed to understand the benefits and potential outcomes of SDM in risk assessment and management, in relation to different types of risk, and the perspectives of stakeholders in different settings such as inpatient and crisis services, and specific sub-groups such as children and young people and older adults.

## Supporting information

S1 Appendix SRQR 21-item checklist. (DOCX)

S2 Appendix Interview schedule. (PDF)

S3 Appendix Feedback from role-playing. (DOCX)

S4 Appendix Coding guide. (DOCX)
