# Supplementary material for: Barriers and enablers to shared decision-making in assessment and management of risk: A qualitative interview study with people using mental health services
Source: PLOS Ment Health. 2024 Nov 13;1(6):e0000157. doi: 10.1371/journal.pmen.0000157 (PMC12798311; doi:10.1371/journal.pmen.0000157)
Supplement: S2 Appendix — (PDF) [file pmen.0000157.s002.pdf]

Shared Decision Making in Risk Assessments and Risk Management

**Hi. My name is XXXX.**<br><br>**Thank you for kindly agreeing to take part in this interview. I am here today to ask you a few questions about how risk and safety are considered in the planning of your care. It should take about an hour at most. There are no right or wrong answers; we just would like to know about your involvement in the process of assessing and managing risk.**

Confidentiality

- Remind the person that they have already given consent to be interviewed and check they are still ok with that.
- They may stop at any time.
- Audio-recording interview.
- Remind them that their name will not be used and they will not be identifiable in any way.
- Check Dictaphone is working.
- Read out code name so that their name can be left out of the interview.

Ok, are you ready to start?
In mental health services, there are concerns about ensuring safety and minimising risk and this is what I would like to explore with you today.

You may find some of the following questions a little uncomfortable to answer. Please take your time and answer freely. Remember that you do not have to answer any questions that make you feel uncomfortable and that you can say as little or as much as you like. Let me know if you need to take a break or stop. Ok, so I will start by asking you:

1. What does the term risk means to you?
   Prompt: Some people may focus on one aspect of risk but it can mean lots of different things. Risk can mean the possibility of any harm to you yourself and/or others; this may include violence, aggression, self-harm, suicide, neglect or relapse. Risk can also mean experiencing side effects from your medication; harassment; stigma; discrimination, vulnerability and even isolation.
2. Have you ever felt vulnerable or at risk?
   Prompt: How? Can you tell me a little bit more about that please?
3. In your opinion, do you think your actions or behaviour have ever made other people feel unsafe? Or at risk? How?
   Prompt: Has anyone ever said to you that your actions or behaviour have made them or other people feel unsafe? What are your thoughts about that? Do you agree?
4. Has anyone ever discussed risk or safety with you? Who? What was discussed?
   Prompt: Carer? Friend? Psychiatrist? Care-coordinator? Nurse? Social Worker? Police?

Thank you. Before I continue, I would like to provide you with some information about risk assessments and risk management plans:

- The purpose of the risk assessment is to identify any risks that may possibly occur to yourself and/or to others.

- The risk management plan is then developed to include a set of actions that can be used to prevent the identified risks from occurring.
- Guidelines say that each step in the process of developing a risk assessment and a risk management plan should be based on shared decision making. This means that you, your carer and the responsible professional should all have the opportunity to be informed, involved and able to contribute to the process.

5. Has anyone ever carried out a risk assessment with you? Crisis plan?
   ***Yes** = Who? What did it include?*
   ***No** = Questions 6*
6. Has anyone ever developed a risk management plan with you?
   ***Yes** = Who? What did it include?*
   ***No** = Question 7*
7. Have you ever been given a copy of your risk assessment? How about your risk management plan?
   ***Yes** = How useful did you find these documents? How often do you refer to them?*
   ***No** = would it have been useful for you to have a copy? Why?*
8. I'm interested in how involved people are in the process of identifying and managing risk.
   Yes = Could you tell me a bit more about how you were involved? **Start TDF questions!**
   No **=** You mentioned that you weren't involved is that correct? **Ask below prompts.**
   - Can you think of any reason why you might not have been involved? Do you think you should have been involved in the assessment and management of your risks? Why?
   - How confident would you have felt about being involved in developing your RA and/or RMP? What would have made you feel more confident?
   - How easy or difficult do you think it would have been for you to be involved in discussions about your risks? Why?
   - Is there anyone else that you think should be involved in identifying and/or managing your risks? Who? Why? (*Prompt: carer, friend, support worker, psychiatrist)*
   - What would have been the benefits of you being involved? What would have been the challenges of you being involved?
   - If you wanted to change the way risk is assessed and managed for you in the future, how would you do this?
   - What would help you be more involved in future risk assessments and risk management plans? Prompt: information, skills, training, or a self-appraisal tool

END OF INTERVIEW!

That's all the questions I have for you. Is there anything else you would like to say that we have not covered? Thank you very much for your time.

Theoretical Domains Framework (TDF) Questions

I am going to ask you a few more questions. Please bear with me if some of the questions appear to be quite similar, we would like to know a bit more detail.

THEORETICAL DOMAIN	QUESTION
**KNOWLEDGE**	1. Are you aware of the risk assessment process?<br>2. What advice or information have you received about your risks? From whom?
**SKILLS**	3. Are there any particular skills that you think are needed to identify and manage risk? What skills?
**SOCIAL /PROFESSIONAL ROLE AND IDENTITY**	4. Do you think you should be involved in identifying and managing your risks? Why?<br>5. Do you think anyone else should be involved in identifying and managing your risks? Who? How?
**BELIEFS ABOUT CAPABILITIES**	6. How easy or difficult is it for you to be involved in identifying and managing your risks?<br>7. How confident do you feel about being involved in identifying your risks?<br>*Prompts: Do you feel you are able to contribute? Do you think your input is influential or makes a difference? What would help you feel more confident?*
**OPTIMISM**	8. How optimistic are you that, in the future, you will be involved in identifying or managing your risks?
**BELIEFS ABOUT CONSEQUENCES**	9. What would happen if you were involved in your risk assessment or risk management plan?<br>*Prompts: What would be the benefits? What would be the drawbacks?*
**REINFORCEMENT**	10. What would make you more likely to be involved in discussions about your risks? Is there anything that would stop you/put you off?
**INTENTION**	11. Do you intend to be involved in identifying and managing your risks in the future? Is there anything that might change that? Would this differ depending on the type of meeting –review meetings, ward rounds

	or discharge meetings?
**GOAL**	12. How important is it for you to be involved in identifying and/or managing your risk? Can you say a little more about that?
**MEMORY, ATTENTION AND DECISION PROCESS**	13. What is going on in your mind when you go to an appointment where you may be discussing your risks?
**ENVIRONMENTAL CONTEXT AND RESOURCE**	14. When you think about how risk is assessed and managed, is there anything about the risk assessment process that stops (or helps) you from being involved?<br>*Prompt: Place, time, the method, the person*
**SOCIAL INFLUENCE**	15. Do other people influence whether or not you are involved in identifying and managing your risks?<br>*Prompts: care-coordinator, staff at the community mental health team, family and friends?*
**EMOTIONS**	16. Do your emotions influence your contribution to identifying and/or managing your risk? How?<br>17. When you think about being involved in identifying and/or managing your risks, how do you feel?
**BEHAVIOURAL REGULATION**	18. If you wanted to be involved, or more involved, in identifying and managing your risks, how would you go about this? Are there any strategies you would use?
**OTHER**	19. Do you think that any of what we've talked about today / your involvement in identifying and managing your risks, would be different if you became mentally unwell?

END

That's all the questions I have for you. Is there anything else you would like to say that we have not covered? Thank you very much for your time.
