# Supplementary material for: Barriers and enablers to shared decision-making in assessment and management of risk: A qualitative interview study with people using mental health services
Source: PLOS Ment Health. 2024 Nov 13;1(6):e0000157. doi: 10.1371/journal.pmen.0000157 (PMC12798311; doi:10.1371/journal.pmen.0000157)
Supplement: S3 Appendix — (DOCX) [file pmen.0000157.s003.docx]

**S3 Appendix. SUGAR’s feedback from role-playing of interview**

| **Feedback** | *Quote* | How was this addressed? |
| --- | --- | --- |
| **For the interviewer to remind participants about their ethical rights, including confidentiality, right not to answer any question that makes them feel uncomfortable and where they could access relevant support** | *“Confidentiality important - say you are not going to talk about it unless issues of safety and concern”*  *“... need to say something about risks of interview, uncomfortable, say don't have to answer questions if feel uncomfortable etc.”*  *“check-in at end if concerned person is low, need to contact someone.”* | To address the feedback, information was provided at the start of the interview, reminding participants that confidentiality will be maintained but was conditional. The interviewer also read out the following statement to reassure participants that they could answer questions freely:  “You may find some of the following questions a little uncomfortable to answer. Please take your time and answer freely. Remember that you do not have to answer any questions that make you feel uncomfortable and that you can say as little or as much as you like. Let me know if you need to take a break or stop. Ok, so I will start by asking you…”.  Participants were reminded of sources of support at the end of the interview, i.e., speak to a care coordinator, family member or mental health support charity |
| **Some questions would benefit from further prompting** | *“may have negative memories, first question may need prompting”*  *“when you asked about taking medication, didn't prompt about side effects”* | The PhD candidate kept mindful of the feedback regarding prompting whilst conducting the actual interviews. |
| **For the definition of risk to incorporate the different risk factors** | *“break down/ group questions about risk, e.g. Suicide, medication, hurting yourself.”* | The definition of risk was amended to incorporate the different types of risks and examples provided, i.e., risk to self, others and risks related to a mental illness diagnosis |
| **Positive comments** | *“nice interview style, good balance of listening and prompting, well done”*  *“liked how you were feeding back and reflecting issues, would there be space to expand upon that in full interview…* | N/A |
