# Supplementary material for: Barriers and enablers to shared decision-making in assessment and management of risk: A qualitative interview study with people using mental health services
Source: PLOS Ment Health. 2024 Nov 13;1(6):e0000157. doi: 10.1371/journal.pmen.0000157 (PMC12798311; doi:10.1371/journal.pmen.0000157)
Supplement: S4 Appendix — (DOCX) [file pmen.0000157.s004.docx]

**S4 Appendix. Coding guide**

**Definition of TDF constructs by domain, and application to mental health setting and the implementation of shared decision-making in risk assessment and risk management**

| **Domain** | **Constructs** | **Application to mental health setting** | **Examples/ Rules** |
| --- | --- | --- | --- |
| 1. Knowledge  (An awareness of the existence of something) | Knowledge (including knowledge of condition /scientific rationale), Procedural knowledge, Knowledge of task environment | Service users’ awareness of the risk assessment and/or risk management process.  Service users’ awareness of receiving advice and information about their risks | - Involved and aware - Lack of awareness, involvement or discussion |
| 2. Skills  (An ability or proficiency acquired through practice) | Skills, Skills development, Competence, Ability, Interpersonal skills, Practice, Skill assessment, Coping strategies | Skills needed in identifying and managing risks |  |
| 3. Professional role and identity  (A coherent set of behaviours and displayed personal qualities of an individual in a social or work setting) | Professional identity, Professional role, Social identity, Professional boundaries, Professional confidence, Group identity, Leadership, Organisational commitment | The extent that the service user believes that they should be involved in their risk assessment and risk management  The extent that the service user believes that others should be involved in their risk assessment and risk management | - The professional’s responsibility |
| 4. Beliefs about capabilities  (Acceptance of the truth, reality, or validity about an ability, talent, or facility that a person can put to constructive use) | Self‐confidence, Perceived competence, Self‐efficacy, Perceived behavioural control, Beliefs, Self‐esteem, Empowerment, Professional confidence | The service user’s confidence in being involved in their risk assessment or risk management  The service user’s confidence in being able to contribute, and that their input is influential | - Confident/Lack of confidence - Difficult - Disagreements - Easy |
| 5. Optimism  (The confidence that things will happen for the best or that desired goals will be attained) | Optimism, Pessimism, Unrealistic optimism, Identity | The service user’s confidence that in the future they will be involved in their risk assessment or risk management |  |
| 6. Beliefs about consequences  (Acceptance of the truth, reality, or validity about outcomes of a behaviour in a given situation) | Beliefs, Outcome expectancies, Characteristics of outcome expectancies, Anticipated regret, Consequents | The service user’s belief about benefits/disadvantages of being involved in their risk assessment or risk management | - Stigma and labelling - Distress or upset |
| 7.Reinforcement  (Increasing the probability of a response by arranging a dependent relationship, or contingency, between the response and a given stimulus) | Rewards (proximal / distal, valued / not valued, probable / improbable), Incentives, Punishment, Consequents, Reinforcement, Contingencies, Sanctions | Factors that encourage/discourage service users’ to be involved in their risk assessment or risk management | - Being open and honest - To be informed, gain knowledge, understanding or insight - To have a voice or say - Value SDM or open dialog |
| 8. Intentions  (A conscious decision to perform a behaviour or a resolve to act in a certain way) | Stability of intentions, Stages of change model, Trans-theoretical model and stages of change | The service user’s intention to be involved in their risk assessment or risk management | - Willing/not willing to be involved |
| 9. Goals  (Mental representations of outcomes or end states that an individual wants to achieve) | Goals (distal / proximal), Goal priority, Goal / target setting, Goals (autonomous /controlled), Action planning (with relation to their intention to implement | The relative importance to service users’ to be involved in their risk assessment or risk management | - To keep myself safe |
| 10. Memory, attention and decision processes  (The ability to retain information, focus selectively on aspects of the environment and choose between two or more alternatives) | Memory, Attention, Attention control, Decision making, Cognitive overload / tiredness | The service user’s thinking before attending an appointment where there may be a discussion about their risks |  |
| 11. Environmental context and resources  (Any circumstance of a person's situation or environment that discourages or encourages the development of skills and abilities, independence, social competence, and adaptive behaviour) | Environmental stressors, Resources / material resources, Organisational culture /climate, Salient events / critical incidents, Person x environment interaction, Barriers and facilitators | The environmental context/situation that may encourage/discourage service users from being involved in their risk assessment or risk management | - Frequency of meeting - Travelling to the service |
| 12. Social influences  (Those interpersonal processes that can cause individuals to change their thoughts, feelings, or behaviours) | Social pressure, Social norms, Group conformity, Social comparisons, Group norms, Social support, Power, Intergroup conflict, Alienation, Group identity, Modelling | The interpersonal relationships/process that may influence the service users involvement in their risk assessment or risk management | - Carer - Power - Therapeutic relationship |
| 13. Emotions  (A complex reaction pattern, involving experiential, behavioural, and physiological elements, by which the individual attempts to deal with a personally significant matter or event) | Fear, Anxiety, Affect, Stress, Depression, Positive / negative affect, Burn‐out | Service users' emotions when involved in risk assessment or risk management  Service users’ emotions that may influence their contribution to risk assessment or risk management | - Capacity or mental wellbeing - Feeling unwell - Anxiety |
| 14. Behavioural regulation  (Anything aimed at managing or changing objectively observed or measured actions) | Self‐monitoring, Breaking habit, Action planning (with relation to monitoring their habits) | Service users’ ability to self-monitor and action plan to be involved in their risk assessment or risk management | - If the service user wanted to be involved or more involved in their risk assessment and risk management in **future**, how would they? - Intervention suggestions |

Cane J, O’Connor D, Michie S: **Validation of the theoretical domains framework for use in behaviour change and implementation research.** *Implementation science* 2012, **7:**37
